# High Voltage Electrical Discharges as an Alternative Extraction Process of Phenolic and Volatile Compounds from Wild Thyme (*Thymus serpyllum* L.): In Silico and Experimental Approaches for Solubility Assessment

**DOI:** 10.3390/molecules25184131

**Published:** 2020-09-10

**Authors:** Marinela Nutrizio, Gianpiero Pataro, Daniele Carullo, Serena Carpentieri, Luisa Mazza, Giovanna Ferrari, Farid Chemat, Mara Banović, Anet Režek Jambrak

**Affiliations:** 1 Faculty of Food Technology and Biotechnology, University of Zagreb, 10000 Zagreb, Croatia; mara.banovic@pbf.hr; 2 Department of Industrial Engineering, University of Salerno, 84084 Fisciano, Italy; gpataro@unisa.it (G.P.); dcarullo@unisa.it (D.C.); scarpentieri@unisa.it (S.C.); maz.luisa@gmail.com (L.M.); gferrari@unisa.it (G.F.); 3 ProdAl Scarl University of Salerno, 84084 Fisciano, Italy; 4 GREEN Team Extraction, Université d’Avignon et des Pays du Vaucluse-INRA, UMR408, 84000 Avignon, France; farid.chemat@univ-avignon.fr

**Keywords:** high voltage electrical discharge, wild thyme, Hansen solubility parameters, COSMO-RS, green extraction, bioactive compounds, aromas

## Abstract

The objective of this study was to evaluate the potential of green solvents for extractions of bioactive compounds (BACs) and essential oils from wild thyme (*Thymus serpyllum* L.) using theoretical and experimental procedures. Theoretical prediction was assessed by Hansen solubility parameters (HSPs) and conductor-like screening model for realistic solvents (COSMO-RS), to predict the most suitable solvents for extraction of BACs. An experimental procedure was performed by nonthermal technology high voltage electrical discharge (HVED) and it was compared with modified conventional extraction (CE). Obtained extracts were analyzed for chemical and physical changes during the treatment. Theoretical results for solution of BACs in ethanol and water, as green solvents, were confirmed by experimental results, while more accurate data was given by COSMO-RS assessment than HSPs. Results confirmed high potential of HVED for extraction of BACs and volatile compounds from wild thyme, in average, 2.03 times higher yield of extraction in terms of total phenolic content was found compared to CE. The main phenolic compound found in wild thyme extracts was rosmarinic acid, while the predominant volatile compound was carvacrol. Obtained extracts are considered safe and high-quality source reach in BACs that could be further used in functional food production.

## 1. Introduction

Nowadays, there is growing interest among consumers in functional food production. Functional foods can be defined as natural dietary items that, besides providing nutrients and energy, have health-promoting, disease-preventing, or medicinal properties [1]. Spices and aromatic herbs have been used for culinary purposes since antiquity, but also in traditional medicine for their potential to improve health [2].

*Thymus serpyllum* L., known as a wild thyme, is a species belonging to the *Lamiaceae* family that grows autochthonously in the Mediterranean area. This plant is rich in polyphenolic compounds and essential oils and shows antiseptic, antioxidative, anthelmintic, diaphoretic, antispasmodic, expectorant, carminative, analgesic and diuretic properties [3,4,5]. In order to produce functional foods from wild thyme, it is important to extract its bioactive compounds (BACs) and essential oils and obtain high-quality natural products. Conventional methods for extraction, like Soxhlet extraction, distillation or maceration, usually have a low production efficiency and are time- and energy-consuming methods that use high amounts of organic solvents and high temperatures. For that reason, some thermally sensitive nutritional compounds, like phenols and antioxidants, may be lost during processing [6].

Usually, the extraction from plant sources is carried out using organic solvents due to their high hydrophobicity. These solvents mostly come from nonrenewable resources and are volatile, flammable and toxic solvents that are not environmentally acceptable. One of the most used solvents is *n*-hexane due to its low polarity, easy removal from the final product and stability [7].

With the goal of overcoming these disadvantages, various green extraction methods have been developed. According to the definition, “green extraction is based on the development of extraction processes designed to reduce energy consumption, while using alternative solvents and renewable natural products, and ensuring a safe and high quality extract/product” [8]. In this line, some environmental directives and legislation are reducing solvent emissions and promoting the use of alternative eco-friendly solvents (EU Directive 1999/13/EC). Examples of green solvents include limonene, pinene, vegetable oils, supercritical CO_2_, ionic liquids, deep eutectic solvents or naturally- based deep eutectic solvents (NaDES) that are produced from eco-friendly and low-cost components and specially designed to be used in extraction processes [9,10]. Water and ethanol are also considered as green solvents that are extensively used as they are the cheapest and most available solvents for extraction processes [11].

The replacement of conventional solvents with the most appropriate green solvents enabling one to improve the extraction efficiency, is generally a costly as well as a time-consuming task. For these reasons, theoretical computational models were developed in order to predict the solute-solvent solubility, enabling researchers to reduce solvent and energy consumption, while maximizing the extraction efficiency [12]. Currently, two predictive models are being used as reliable tools to select the most suitable solvents for extraction of target BACs, namely the Hansen solubility parameters (HSPs) that explain the dissolution behavior of solvents and solutes, and the conductor- like screening model for realistic solvents (COSMO-RS) that is a statistical thermodynamics approach based on the results of quantum chemical calculations that provide explanations of the mechanisms of dissolution [13,14].

Within the green extraction principles, various innovative technologies, such as high voltage electrical discharge (HVED), were developed in order to improve the extraction efficiency while reducing solvent, energy, and hazards, and accordingly impact less to the environment [15]. Specifically, HVED is a nonthermal technology based on the phenomenon of electrical breakdown in liquids, which induces physical (e.g., shock waves) and chemical (e.g., formation of free radical species) processes. During the treatment, a gas is flown in the reactor resulting in gas ionization, and accordingly, cold plasma is being formed. These phenomena affect the plant cell, by inducing cell disintegration, a process where electric field is increasing the permeability of the cell membrane, resulting in membrane pores and enhancing the release of intracellular components [16]. Several extractions of bioactive compounds from plant materials were reported with HVED, such as peanut shells [17], pomegranate peel [18], sesame cake [19], grapefruit peels [20], and orange peel [21]. However, to date, no literature data was found on HVED-assisted extraction of valuable compounds from wild thyme.

Obtained plant extracts could be used for functional food production, but also in other spheres of food industry, as colorants, preservatives and other different kind of additives [22,23]. Furthermore, plant extracts could be further preserved by encapsulation methods for controlled release of BACs [24] in food industry [25], but in other industries as well, such as cosmetics [26] or agriculture [27], and in various fields of pharmaceutical industry and medicine due to plants’ natural biological properties [28,29].

The aim of this study was to evaluate the potential of green solvents for extraction of BACs and essential oils from wild thyme. Both theoretical and experimental analysis were performed. For theoretical analysis, HSPs and COSMO-RS software were used. Further experimental analysis was performed by means of HVED as a green nonthermal technology to extract BACs and volatile compounds from wild thyme in comparison with modified conventional extraction (CE).

## 2. Results

### 2.1. Theoretical Prediction

The solubility of wild thyme BACs in different green solvents was evaluated by theoretical models using HSPs and COSMO-RS software. Results are presented as comparison with the conventionally used solvent *n*-hexane (first column). Further green solvents chosen for analysis were ethyl acetate, methyl acetate, ethyloleate, ethanol, 1-butanol, isopropanol, methanol, limonene, α-pinene, cymene, β-myrcene, cyclopentyl methyl ether (CPME), dimethylcarbonate (DMC), 2-methyltetrahydrofuran (MeTHF) and water.

Assessment with HSPs allowed relative energy difference (RED) results (Table 1), the estimation of solvent capacity to dissolve solutes. RED values < 1 represent very good solubility (green color); 1–3 presents medium solubility (yellow color); while results > 3 present poor solubility > 3 (red color). Results showed that water showed low solubility for all assessed wild thyme compounds. On the other side, cyclopentyl methyl ether (CPME) showed highest potential for extractions from wild thyme and very good solubility for carene, α-thujene, α-terpinene, δ-terpinene, borneol, α-cadinol, linalool, piperitone, β-caryophyllene, copaene, α-cubebene, β-elemene, β-cadinene, δ-cadinene, isospathulenol, α-tocopherol, and ascorbic acid.

The COSMO-RS software integrates a quantum chemistry approach that allows the calculation of various properties such as the relative solubility of a compound in several solvents [30]. Results of probability of solubility assessed by COSMO-RS theoretical prediction are given in Table 2. Results from 0–20% present low probability of solubility (red color); 20–60% medium probability of solubility (yellow color); and 60–100% high probability of solubility (green color). Water was presented as a solvent with lowest potential to solvate BACs from wild thyme, however, it is expected that water will have high potential for solubility of quinic acid, caffeic acid and ascorbic acid. The best solubility of BACs from wild thyme was presented with MeTHF where all evaluated compounds showed a high probability of solubility.

**Figure 1:**
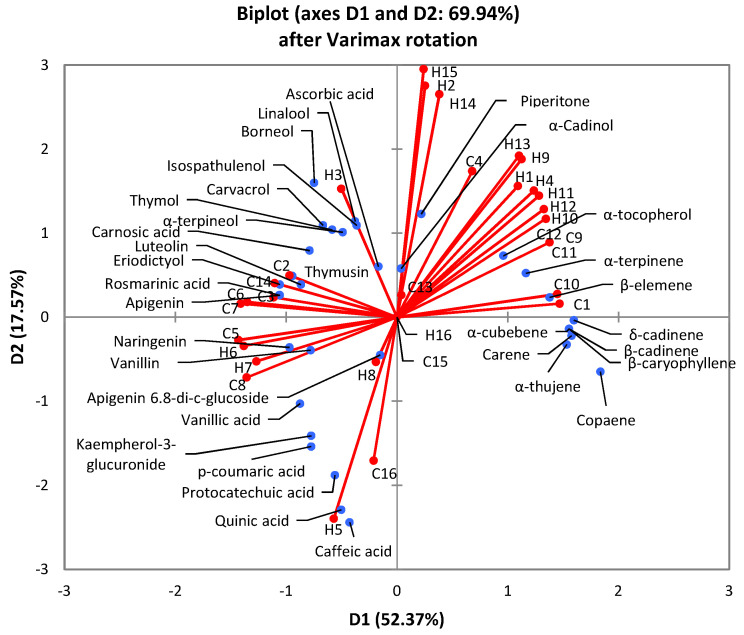
Biplot of the principal component analysis applied on data based on theoretical prediction models including (i) evaluated compounds (marked blue) and (ii) Hansen solubility parameters and COSMO-RS parameters (marked red). H-Hansen solubility parameters, C-COSMO-RS parameters, 1—*n*-hexane, 2—ethyl acetate, 3—methyl acetate, 4—ethyloleate, 5—ethanol, 6—1-butanol, 7—isopropanol, 8—methanol, 9—limonene, 10—α-pinene, 11—cymene, 12—β-myrcene, 13—cyclopentyl methyl ether, 14—dimethylcarbonate, 15—2-methyltetrahydrofuran, 16—water.

The qualitative modelling using principal component analysis (PCA) to identify potential grouping and correlations between HSPs and COSMO-RS results was performed. PCA graph including both components and HSPs and COSMO-RS variables is given in Figure 1.

### 2.2. Experimental Procedure

Extracts obtained by CE and HVED were analyzed for changes during the extraction, therefore, physical and chemical parameters of extracts were measured. Physical parameter results, including pH, conductivity, temperature and power used for HVED treatment, are given in Table 3. Also, cell disintegration index (Z_p_) was calculated.

**Figure 2:**
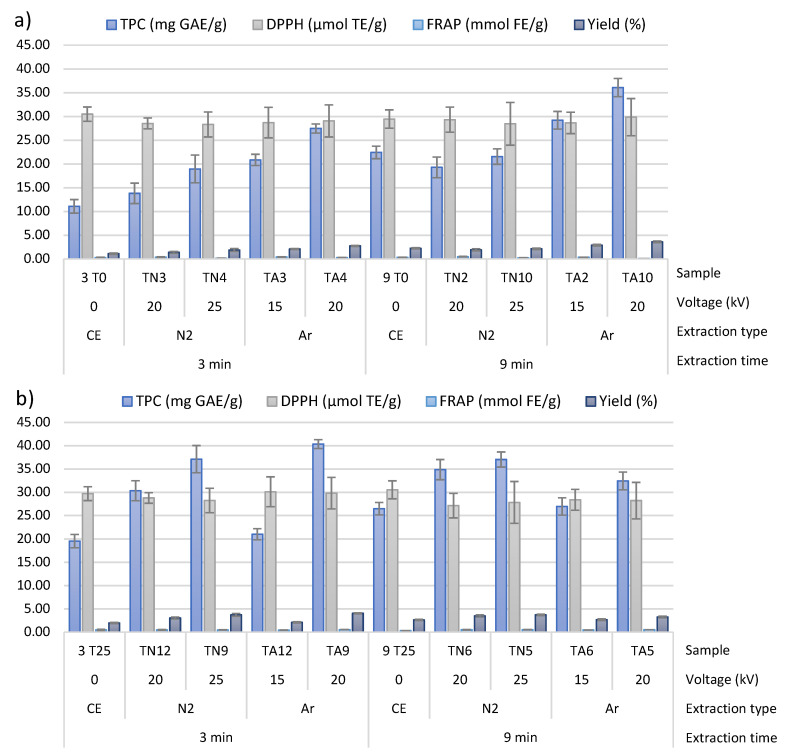

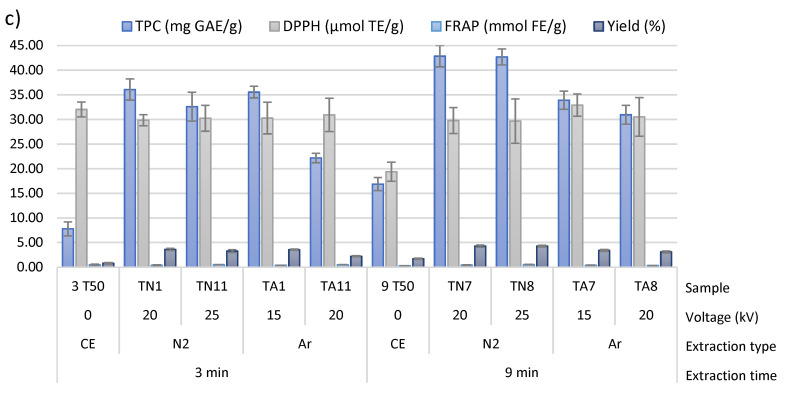
Determination of bioactive compounds—total phenolic compounds (TPC) values, antioxidant activity (DPPH, FRAP) and yield—measurements for CE and HVED treated samples when: (**a**) water was used as a solvent, (**b**) 25% ethanol was used as a solvent, (**c**) 50% ethanol was used as a solvent. Extraction type: CE—conventional extraction, N_2_—HVED extraction with nitrogen, Ar-HVED extraction with argon.

Furthermore, chemical analyses of wild thyme extracts were performed. In order to compare extraction efficiency of BACs by means of HVED, compared to CE, obtained extracts were analyzed by spectrophotometric methods—total phenolic content (TPC) and antioxidant activity (2,2-diphenyl-2-picrylhydrazyl (DPPH) free radical assay and ferric reducing antioxidant power (FRAP) assay). Results are given in Figure 2. Extraction yields are presented as g GAE/g of sample × 100 and expressed as percentages (%).

In order to quantify individual phenolic compounds, an ultra performance liquid chromatography-tandem mass spectrometry characterization (UPLC-MS/MS) analysis was performed. Wild thyme extracts were analyzed for apigenin, carnosol, diosmetin, hydroxytyrosol, luteolin, oleanolic acid, quercetin, rosmarinic acid, p-cymene, thymol, carvacrol and camphor, results are given in Table 4.

The analysis of volatile compounds from wild thyme extracts was performed by HS-SPME/GC-MS method. Analyzed compounds included 1,8-cineol, linalool, thymol and carvacrol, and results are shown in Table 5.

Statistical analysis was performed in in XLStat (MS Excel 2010). An analysis of covariance (ANCOVA) was used to analyze the impact of independent variables: treatment time, voltage, ethanol content and treatment type (CE, HVED—nitrogen or HVED—argon) to measured physical and chemical parameters. For UPLC-MS/MS and GC-MS results, all individual (data not shown) and total of all compounds was assessed. The p-values present the statistical significance of each of the factor and it was significant at *p* ≤ 0.05, and are shown as bolded in Table 6.

Furthermore, in order to assure safe and high-quality final product, residue levels of pesticides and metals has been analyzed. At the moment, trace content of metals and pesticides in foods are regulated via European Commission Regulations No 1881/2006 and No 396/2005, respectively. Regulations for dietary supplements were chosen as the nearest category to evaluate health safety requirements for wild thyme and provide maximum residue levels (MRLs). Results of analyzed pesticides levels in dried wild thyme is given in Table 7.

Results of residue levels of metals found in dried wild thyme and selected extracts are given in Table 8. Due to possible abrasion of electrodes during the treatment with HVED, content of metals was analyzed also in wild thyme extract obtained by HVED. For this purpose, extract TN8 was chosen that was treated with highest voltage (25 kV), nitrogen, for longer time (9 min) and highest ethanol content (50%), since highest abrasion is expected in such extract.

## 3. Discussion

The extraction of BACs and volatile compounds from wild thyme was assessed by theoretical and experimental procedures. Theoretical prediction was calculated by computational simulation methods using HSPs and COSMO-RS software, while experimental analysis was done by HVED as a nonthermal extraction technology. For better comparison, both methods were performed at room temperature.

### 3.1. Theoretical Prediction of Solubility of Wild Thyme Compounds in Green Solvents

#### 3.1.1. Wild Thyme Compounds Solubility in Green Solvents by HSPs

The solubility of wild thyme compounds in selected green solvents assessed by HSPs is given in Table 1 and presented as a comparison with conventionally used *n*-hexane (first column). Results are shown as a RED value that estimates the potential of a solvent to dissolve solutes. RED values from 0–1 present very good solubility, medium solubility is 1–3, and poor solubility > 3. It is clear that some alternative (green) solvents have better solubility then n-hexane and for that reason, it is not the best solvent for extraction of wild thyme BACs from theoretical perspective. According to solvent with highest number of very good soluble compounds, followed by medium and poor solubility, the order of solvents that could be used for better solubility of wild thyme compounds was as following: CPME > limonene > ethyloleate > cymene > β-myrcene > MeTHF > α-pinene > *n*-hexane > ethylacetate > DMC > methylacetate > 1-butanol > isopropanol > ethanol > methanol > water. It can be concluded from the results that wild thyme compounds have the lowest solubility in water, followed by primary alcohols (ethanol, 1-butanol and methanol) and secondary alcohol isopropanol. On the other side, these solvents are the cheapest green solvents that is the great advantage for their usage. CPME showed the highest theoretical prediction for solution of wild thyme compounds.

A classical rule “like dissolves like” can be applied to theoretical prediction for solvent-solute solubility. According to this rule, more polar solvents will have higher probability of solubility for polar compounds, while non-polar solvents will have a tendency to dissolve less polar or non-polar solutes. This was shown with HSPs results, where non-polar (or weakly polar) solvents like n-hexane, limonene, CPME, methylacetate, ethyloleate, cymene, β-myrcene and α-pinene had higher probability of solubility of less polar compounds such as hydrophobic thymol, α-thujene, piperitone and other.

#### 3.1.2. Wild Thyme Compounds Solubility in Green Solvents Assessed by COSMO-RS Software

The COSMO-RS software was also used for evaluation of solubility of wild thyme compounds in selected green solvents. Results are presented in probability of solubility (%) where low probability is marked with red color, medium probability with yellow color, and high probability of solubility with green color. Similar trend was shown as with HSPs, but higher potential for solution of solutes was predicted with COSMO-RS software. Results showed that MeTHF has the highest probability of solubility of wild thyme compounds, and that all solvents, except water, have higher potential for solution during extractions with wild thyme, compared to *n*-hexane. Solvents are ranked as follows: MeTHF > CPME > ethyl acetate > methyl acetate > 1-butanol > ethyl oleate > isopropanol > ethanol > methanol > DMC > limonene > cymene > β-myrcene > α-pinene > *n*-hexane > water.

These results show the solubility of each individual compound in the solvent, but the extraction does not depend only on solubility, but also on the quantity of each compound in the plant. Therefore, experimental analysis was performed. Taking into consideration results obtained by two theoretical prediction methods, it was decided to perform extraction using green solvents with potential to replace *n*-hexane: water, ethanol, limonene, α-pinene, DMC and ethyl acetate. Since no electrical discharge was achieved with limonene, α-pinene, DMC and ethylacetate, for further analysis, only water and aqueous ethanol 25% and 50% (*v*/*v*) were taken.

#### 3.1.3. Principal Component Analysis (PCA) of Theoretical Prediction Results

The PCA analysis was conducted in order to correlate the results from HSPs and COSMO-RS software. The biplot of PCA assessment is given in Figure 1. A total of 52.37% of variance was clarified in the observed data set. The biplot showed that analysed compounds have spread over all quadrants, while HSPs and COSMO-RS parameters were placed in first, second and third quadrants. A grouping of selected compounds from the same group (i.e., oxygenated monoterpenes, sesquiterpenes, flavonons, etc.) is notable, where all compounds from the same group were grouped in the same quadrant or at least, at the same side of *x* or *y* axis, except monoterpenes that were placed in first, second and fourth quadrants. Additionally, grouping of solvents was also notable. For example, short chain alcohols (ethanol, methanol, 1-butanol and isopropanol) were placed all in the third quadrant according to HSPs and in second and third quadrants according to COSMO-RS results. All monoterpenes, regarding the analysed method, were placed in the first quadrant of the PCA biplot. 

It is clear that neither of solvents showed completely different results with two theoretical prediction models, i.e., no solvents were placed in inversely proportional quadrants (I-III or II-IV). Furthermore, most solvents were placed in the same quadrant with HSPs and COSMO-RS prediction including *n*-hexane (1), methyl acetate (3), ethyl oleate (4), ethanol (5), methanol (8), limonene (9), α-pinene (10), cymene (11), β-myrcene (12), and CPME (13). For that reason, it can be concluded that similar data are given with HSPs and COSMO-RS results, although some differences could be noted, especially with higher probability of solution given with COSMO-RS data (Table 2).

### 3.2. Experimental Assessment of Extraction of Wild Thyme Bioactive Compounds by Means of High Voltage Electrical Discharge (HVED)

#### 3.2.1. Physical Properties of Wild Thyme Etracts Obtained by HVED Compared to Conventional Extraction 

The aim of the experimental analysis was to extract BACs from wild thyme by HVED extraction. During the HVED treatment, a gas is flowing through the needle (electrode) and it is being ionized forming a cold plasma. The type of gas used in the treatment since different gas ionize at different voltages and different radical species are being formed during discharge. Therefore, different results could be obtained with different gases [31]. For that reason, it was difficult to obtain electrical discharges with nitrogen under 20 kV so 20 and 25 kV were chosen for nitrogen treatments, while lower voltages of 15 and 20 kV were chosen for argon treatments.

Results of physical parameters, including pH, conductivity, temperature before and after the treatment and power used during HVED treatment, are given in Table 3. pH of wild thyme extracts ranged from 5.12 ± 0.18 to 6.31 ± 0.15 meaning that all extracts were slightly acidic, and no significant changes in pH were noted between HVED extracts and extracts obtained by CE. Electrical conductivity was in range from 89.0 ± 6.3 μS/cm (extract TA11) to 646.0 ± 30.1 μS/cm (extract TA2). Statistical analysis of influence of main effect (treatment time, voltage, ethanol content and treatment type) to results of physical parameters is shown in Table 6. Statistically significant influence (*p* ≤ 0.05) to pH and conductivity had only ethanol content. With higher ethanol content, pH increased, while conductivity decreased. Regarding the temperature of extracts, it was shown that the maximum measured temperature after the HVED treatment was 37.8 ± 1.3°C. For statistical analysis, a temperature difference calculated as a difference after and before HVED treatment, was taken in account for statistics. Maximum temperature difference was 12.2 °C for sample TA2 treated for 9 min with argon at 15 kV and water as a solvent, where also the highest conductivity was noted. Temperature difference was higher with longer treatment time, higher voltage, argon and lower ethanol content, while statistically significant influence was noted for all parameters except voltage. Regarding the power used for HVED treatment, statistically significant influence of treatment time and voltage was observed, where longer treatment time and higher voltage increased power. This trend was statistically confirmed for treatment time and voltage.

Additionally, Z_p_ was used to select the optimal HVED treatment conditions where the highest cell membrane permeabilization degree was achieved. According to results (Table 3), the highest permeabilization happened in extract TA2 (9 min, 15 kV, 0% of ethanol), sample where highest conductivity and highest temperature difference was noted, indicating the highest yield of extraction, although it was not proven with results of phenolic compounds and antioxidants (Figure 2). Z_p_ lowered with higher ethanol content and increased with higher voltage applied. These results indicate potential use of Z_p_ for assessment of electroporation during HVED treatment. However, statistical data showed that only ethanol content had a significant influence to the electrical conductivity, and Z_p_ accordingly. Therefore, no clear conclusions could be provided regarding Z_p_ index. 

#### 3.2.2. Effect of HVED-Assisted Extraction on Recovery of Bioactive Compounds from Wild Thyme 

BACs are generally used in various industries such as food, cosmetic and pharmaceutical industries, especially due to the their antioxidative, antiseptic and antitumor capacity [32]. Extraction of BACs from wild thyme was performed by HVED as a green extraction technology in order to obtain high-quality extracts reach in BACs that could be used for functional food production. The obtained extracts were analyzed for total phenolic content and antioxidant activity (DPPH and FRAP) and compared with modified CE (Figure 2).

HVED extraction showed higher results for all measured methods including TPC, DPPH and FRAP. The highest content of TPC was found in extract TN7 that was extracted using HVED, nitrogen at 20 kV, and with 50% ethanol (42.86 ± 2.38 mg GAE/g, Figure 2c). The highest antioxidant activity was found in extracts obtained by HVED using argon: TA7 and TA9 for DPPH and FRAP methods, respectively. Better extraction of polyphenols and antioxidants was enhanced generally with longer treatment time, higher ethanol content and higher voltage. TPC and DPPH were higher for treatment with nitrogen than argon, while FRAP values were higher for argon treatment. In order to define the best extraction parameters, it is necessary to establish the amount of BACs extracted according to the defined experimental design. Statistically significant influence of ethanol content was noted for TPC and FRAP, while longer treatment time significantly influenced to DPPH results (Table 6). Based on the model, the extraction yield was expressed as percentage of extracted polyphenols per g of sample (g GAE/g of sample). Therefore, yield of extraction was in line with results of TPC and the same trend was noted. The highest yield of extraction was seen for extract TN7 extracted with HVED using nitrogen for 9 min, at 20 kV with 50% of ethanol. In average, HVED extraction showed 2.03 higher yield of extraction compared to CE under same extraction conditions (ethanol content and time of extraction), and it was 2.07 times higher for nitrogen and 1.99 times higher for extraction with argon. A correlation analysis between TPC, DPPH and FRAP showed that the highest correlation was found between TPC and FRAP (0.259). Although these correlations were not high, all methods showed positive correlations. This is not an unusual occurrence since each of this method is based on different mechanism and is performed in different conditions.

Jovanović et al. measured TPC in wild thyme extracts after extraction with 1:30 solid-to-solvent ratio and 50% ethanol. With these conditions, results were 26.6, 29.8 and 32.7 mg GAE/L for extraction by maceration, heat and ultrasound-assisted techniques, respectively [5]. These results are much lower compared to HVED, since extract TN7 that had the highest TPC (42.86 ± 2.38 mg GAE/g) had the equivalent of 214.31 mg GAE/L. Đukić et al. evaluated various conventional (Soxhlet and macerate extraction) and non-conventional extraction (ultrasound, microwave and subcritical water extractions) methods from wild thyme. The results showed that the highest TPC and antioxidant activity was found using subcritical water extraction (141.12 ± 0.23 mg GAE/g and 170.32 ± 0.87 mg AA/G) [33]. Although these results seems higher compared to our study, no conclusions could be made regarding the best extraction method since different analytical methods were used and different solvents with different solvent to solid ratio were used.

#### 3.2.3. Effect of HVED on Phenolic Composition of the Extracts

Results of the performed UPLC-MS/MS analysis from wild thyme extracts have shown (Table 4) that the main compounds in wild thyme extracts were in the following order, according to their average content in all extracts: rosmarinic acid, oleanolic acid, luteolin, apigenin, diosmetin, hydroxytyrosol, quercetin, carnosol, camphor, thymol, *p*-cymene, and carvacrol. Rosmarinic acid was found to be the main phenolic compound found in wild thyme extracts, which was already confirmed by Jovanović et al. [5]. Rosmarinic acid was found in higher concentrations in HVED extracts compared to CE and it was higher with higher concentrations of ethanol, up to 50% in extracts. Chromatograms of representative extracts obtained by CE (Figure S1) and HVED (Figure S2) present the main components found by UPLC-MS/MS and their differences. The most pharmacological importance of rosmarinic acid is its antioxidant and anti-diabetic properties [34]. For that reason, wild thyme extracts are a valuable source for functional food production. Apigenin, carnosol, luteolin, oleanolic acid, quercetin, rosmarinic acid and camphor were found to be statistically significant higher with higher ethanol content. Also, luteolin and cymene significantly depended on treatment type. The sum of all BACs measured by UPLC-MS/MS was compared with results of TPC and correlation of 0.384 (Table S1) was found between UPLC-MS/MS results and TPC, which is the highest correlation compared to DPPH and FRAP.

Experimental results have confirmed theoretical results obtained by HSPs and COSMO-RS software. Apigenin, luteolin and rosmarinic acid were confirmed to be better soluble with higher ethanol content of the solvent, which was also indicated by the theoretical results (Table 1 and Table 2). Although theoretical results showed better solubility of these compounds in ethanol, compared to water, still poor solubility was expected in both solvents calculated by HSPs (Table 1). Low probability of solubility (red color, Table 2) was also expected by COSMO-RS prediction in water. However, for apigenin, luteolin and rosmarinic acid, a very high probability (100%) was predicted for solution in ethanol. Therefore, it can be concluded that experimental results confirmed theoretical results regarding higher solubility of wild thyme compounds in ethanol compared to water. On the other hand, better results are given with COSMO-RS software than HSPs since high concentrations of apigenin, luteolin and especially rosmarinic acid were found after extraction with HVED.

#### 3.2.4. Effect of HVED on the Content of Volatile Compounds in Wild Thyme Extracts

Results of HS-SPME/GC-MS analysis of volatile compounds from wild thyme extracts are given in Table 5. Carvacrol and thymol were already measured by UPLC-MS/MS, but very low concentrations were found by this methods (< 3 ng/mL), so these volatile compounds were measured also by HS-SPME/GC-MS. Results showed that monoterpenoid phenol carvacrol (0.47–21.86%) was the predominant volatile compounds found in wild thyme extracts, followed by linalool, thymol, and finally, 1,8-cineole that was found in small amounts (0.96–3.46%). A similar trend was found in a study where wild thyme essential oil was extracted by supercritical carbon dioxide as another green extraction method, and carvacrol and thymol were found as main compounds in the extract. That study demonstrated that oil rich fraction of the CO_2_ extract yields were 0.3–0.5% [35]

All measured compounds were found in higher concentrations in HVED extracts, compared to CE. 1,8-cineol and linalool were higher in HVED extracts obtained with nitrogen, while thymol and carvacrol were higher in extracts where argon was used. The differences between CE and HVED treated extracts in the same extraction conditions are presented in Figure S3a–c. The influence of ethanol content was difficult to assess, since overlapping of peaks profile happened with ethanol peak in chromatograms. For that reason, most extracts containing ethanol do not have available data for compound concentration. However, a traceability of results is visible in HVED extracts with nitrogen (RN2-RN6 and RN9-RN10) and argon (RA2-RA6 and RA9-RA10), except for linalool that was found in extracts MA5 and MA6, while it was not found in extracts MN5 and MN6. Similar results were presented in study by Sonmezdag et al., where thymol and carvacrol were found to be the main components of essential oil of wild thyme, assessed by GC-MS [36].

Results of thymol, carvacrol and linalool were compared with theoretical prediction models where low solubility was expected in water measured with both methods (HSPs and COSMO-RS) and in ethanol when calculated by HSPs. However, very good solubility of carvacrol and thymol (100%) was expected in ethanol, while medium solubility was expected for linalool (52.48%) evaluated by COSMO-RS software. Since it was difficult to evaluate experimentally extracts containing ethanol, no clear correlations can be drawn with theoretical results. On the other hand, COSMO-RS results showed lower probability of solubility of linalool, compared to thymol and carvacrol, which was proven with experimental results.

#### 3.2.5. Determination of Pesticides and Heavy Metals in Wild Thyme Samples

High levels of pesticides and metals in food could cause serious toxicological effects. For that reason, it is important to monitor level of pesticides and metals even in raw material, in order to assure high-quality final product. The results of pesticides from dried wild thyme are given in Table 7. Residue levels of pesticides were determined according to EC Regulation No 396/2005. This Regulation include EU Pesticides database with all active substance of pesticide (EC 1107/2009) and their MRLs (EC 396/2005). Results showed that residue levels of all pesticides measure in wild thyme were lower than limit of quantitation of the method. For that reason, no exact data are provided. For most results, limit of quantification was below MRL and for other data it was not possible to quantify exact level and analyze if it was below MRL.

Residue level of heavy metals was determined according to EC Regulation No 1881/2006. Results showed that levels of heavy metals lead (Pb), cadmium (Cd) and mercury (Hg) in dried wild thyme sample were below MRLs for each substance (Table 8). Further analysis of safety and quality included analysis of other metals (chromium (Cr), nickel (Ni), manganese (Mn), iron (Fe), copper (Cu) and zinc (Zn)). These metals were measured in dried wild thyme material and in HVED extract (TN8). For these metals, no MRL data is provided because these are not included in EC Regulations. Although results are given for dried wild thyme per kilogram of plant and for extract per kilogram of final extract, it is clear that levels of Ni, Mn, Fe, Cu and Zn decreased, while level of Cr significantly increased. It could have happened because of abrasion of electrodes during HVED treatment and release of metals, such as Cr, in a solvent. However, it is possible to obtain safe and high-quality final extract of wild thyme since dried plant is considered as safe for human use. Detailed analyzes should be performed regarding levels of metals released in extracts during HVED treatment.

## 4. Materials and Methods 

### 4.1. Theoretical Prediction

#### 4.1.1. Hansen Solubility Parameters (HSPs)

HSPs is a method for characterization of solute-solvent interactions according to the classical “like dissolves like” rule. A detailed concept of HSPs is described in paper by Aissou et al. [30]. For solvent optimization, a simple composite affinity parameter, the RED number, has been calculated to determine the solubility between solvents and solutes:

(1) $$\mathrm{RED}=\frac{R_{a}}{R_{o}}$$

where R_o_ is the radius of a Hansen solubility sphere and R_a_ is the distance of a solvent from the center of the Hansen solubility sphere. The Hansen solubility sphere is determined by three Hansen parameters: dispersion (δ_d_), polar (δ_p_) and hydrogen bonding (δ_h_) where R_0_ determines the radius of the sphere in Hansen space and its center is the three Hansen parameters.

A potentially good solvent has RED number smaller than 1 (the compound has similar properties and will dissolve), while medium and poor solvents have RED values of from one to three and more than 3, respectively. The chemical structures of the solvents and solutes discussed in this article could be mutually transformed by JChemPaint version 3.3 (GitHub Pages, San Francisco, CA, USA) to their simplified molecular input line entry syntax (SMILES) notations, which were subsequently used to calculate the solubility parameters of the solvents and compounds (HSPiP Version 4.0, Hansen Solubility, Hørsholm, Denmark).

#### 4.1.2. Conductor-Like Screening Model for Real Solvents (COSMO-RS) Software

**Figure 3:**
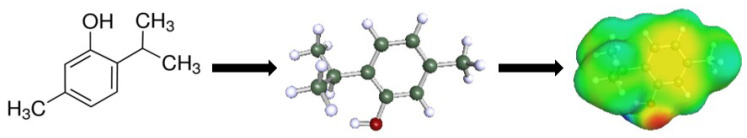
Chemical structure of thymol and translation to sigma surface in COSMO-RS software. The red zone represents the negative charge density of the molecule and the positive values of the sigma, green zones represent non-polar surface of the molecule.

The COSMO-RS software is a statistical thermodynamic method for molecular description and solvent screening based on a quantum-chemical approach [37]. The prediction is based on a two-step procedure—microscopic and macroscopic. The procedure was explained in details by Aissou et al. [30]. The COSMOthermX program (version C30 release 13.01) was used to calculate the relative solubility between the solid compound and the liquid solvent in terms of the logarithm of the solubility in mole fractions (log_10_(x_solub_)). The logarithm of the best solubility was set to 0 and all other solvents were given relative to the best solvent. Also, the logarithm was transformed into probability of solubility (%). The calculation was performed at room temperature (20 °C). As an example, Figure 3 depicts the molecular structure of thymol and its sigma surface.

### 4.2. Experimental Procedure

#### 4.2.1. Plant Materials

Dried wild thyme (*Thymus serpyllum* L.) material was used for extractions. It was provided by local specialized herb store (Suban d.o.o., Samobor, Croatia). Plant material was collected during the flowering season in 2017, in the northwestern part of Croatia, dried naturally, and stored in polyethylene bags in a dark and dry place, at ambient temperature until extractions. Measured plant particle size distribution was measured by the laser particle size analyzer Mastersizer 2000 (Malvern Instruments GmbH, Herrenberg, Germany) and results were as following: d(0.1) ≤ 158.4 μm; d(0.5) ≤ 289.0 μm; d(0.9) ≤ 457.4 μm. All extractions were performed using 1 ± 0.01 g of herb material added to 50 mL of extracting solvent (distilled water, 25% and 50% aqueous ethanol (*v*/*v*)) based on preliminar study results were different solvent to herb ratios were used and higher range of ethanol content.

#### 4.2.2. High Voltage Electrical Discharge (HVED) and Conventional Extraction

For high voltage electric discharge generation, a IMP-SSPG-1200 generator (Impel group d.o.o., Zagreb, Croatia) was used previously described in more detail by Nutrizio et al. [38]. Previously optimized extraction parameters were set to frequency of 100 Hz, pulse width of 400 ns, voltage of 15 and 20 kV for argon gas and 20 and 25 kV for nitrogen gas, and treatment time of 3 and 9 min. The extraction was performed in a 100 mL beaker shaped reactor where the herb-mixture was transferred. The gap between electrodes was 15 mm. Argon and nitrogen gases were flowed in through the needle with the flow 0.75 L min^−1^. Power used during the HVED treatment was measured directly from the HVED instrument. A modified conventional extraction (CE) method was performed for comparison purposes, with same extraction conditions as HVED: at (room) temperature by dissolving the dried thyme material (1 g) in the solvent (50 mL) with light magnetic stirring during 3 or 9 min. Both extractions, HVED and conventional, were performed in duplicates.

#### 4.2.3. Physical Properties of Wild Thyme Extracts 

The pH and electrical conductivity of extracts were measured immediately after HVED treatment using a pH and conductivity meter HI-2030-edge (Hanna Instruments, Bedfordshire, UK). Temperature was measured using an infrared thermometer PCE-777 (PCE Instruments Ltd., Southampton Hampshire, UK). 

Electrical conductivity measurements of the extracts were used to quantify the degree of cell permeabilization induced by HVED treatment of given intensity. The results are presented as cell disintegration index (Z_p_) and are calculated according to literature data for pulsed electric fields [39] with some modifications, expressed as follows:

(2) $$Z_{p}=\frac{\sigma_{t}-\sigma_{i}}{\sigma_{d}-\sigma_{i}}$$

where σ_t_ is the actual measured conductivity value of the extract, σ_i_ is the conductivity of the extract obtained from untreated samples (intact cell tissue), while σ_d_ is the highest value of conductivity, related to the maximally damaged cell tissue. For each treatment condition investigated the Z_p_ value ranged between 0 (for intact tissue) and 1 (for fully permeabilized tissue).

#### 4.2.4. Determination of Total Phenolic Content (TPC)

TPC of thyme extracts was determined using Folin-Ciocalteu method as previously described [38]. The calibration curve was prepared using 50 to 500 mg/L of gallic acid in ethanol. The concentration of TPC was expressed in mg of gallic acid equivalents per gram of sample (mg GAE/g of sample). All measurements were performed in duplicates.

#### 4.2.5. Determination of Antioxidant Capacity

##### 2,2-Diphenyl-2-Picrylhydrazyl (DPPH) Free Radical Assay

DPPH assay of thyme extracts was determined according to previously reported procedure [38]. The results were calculated using calibration curve for Trolox and expressed as µmol of Trolox equivalents per gram of samples (µmol TE/g of sample).

##### Ferric Reducing Antioxidant Power (FRAP) Assay

The FRAP assay was conducted as previously reported [40]. FRAP values were calculated according to the calibration curve for FeSO_4_·7H_2_O and expressed as µmol of Fe^2+^ equivalents (FE) per gram of sample (µmol FE/g of sample).

#### 4.2.6. Ultra Performance Liquid Chromatography-Tandem Mass Spectrometry Characterization of Phenolic Compounds (UPLC-MS/MS)

Method for UPLC-MS/MS (Expert Ultra LC 110, Eksigent, Redwood city, CA, USA; 4500 QTRAP SCIEX, Redwood city, CA, USA) reference conditions [41] were conducted using a Luna Omega 3 μm Polar C18 100 Å, 100 × 4.6 mm column (YMC America, Inc., Allentown, PA, USA) at a thermostatted column temperature of 40 °C, automatic sampling temperature 4 °C, and injection volume 10 μL using the original wild thyme extract concentration obtained by CE and HVED. Mobile phases consisted of: A 100% H_2_O with 0.1% HCOOH (*v*/*v*) and B 100% acetonitrile with 0.1% HCOOH (*v*/*v*) with mobile phase flow 0.40 mL/min. Gradient was set as follows: 1 min 10% B, 2 min 10% B, 15 min 90% B, 25 min 90% B, 27 min 10% B, 30 min 10% B. Determination conditions for MS/MS detector were: ionization -negative ionisation mode atmospheric pressure - negative ionization at atmospheric pressure; ionization temperature: 500 °C, i.e., gas temperature combining the mobile phase at the exit from the capillary before ionization; curtain gas 30 psi, temperature of 500 °C, ion souce gas 1–50 psi, ion souce gas 2–55 psi. Voltage on the electrode after capillary and next to ionization (ion spray voltage) was −4500 V. Limit of detection for all compounds was 0.10 ng/mL. A post-acquisition data processing was done in MultiQuant 3.0.2. Software, v3.022950.0, 2015.

#### 4.2.7. Headspace Solid-Phase Microextraction (HS-SPME) Followed by Gas Chromatography and Mass Spectrometry Analysis (GC-MS)

HS-SPME was performed with manual SPME holder using three fiber covered with DVB/CAR/PDMS (Supelco Co., Bellefonte, PA, USA). For HS-SPME, the finely samples 2 mL were placed separately in 10 mL glass vials and hermetically sealed. The vials were maintained at 60 °C during equilibration (15 min) and extraction (45 min). Thereafter, the SPME fiber was withdrawn and inserted into GC-MS injector (250 °C) for 6 min for thermal desorption. The procedure was similar as reported [42]. GC-MS analyses were done on an Agilent 7890A Gas Chromatograph (Agilent Technologies, Palo Alto, CA, USA) equipped with a mass spectrometer (MSD) model 5977E (Agilent Technologies) and HP-5MS capillary column (5% phenyl-methylpolysiloxane, Agilent J & W). The GC conditions were same as reported previously by Jerković et al. (2016). The oven temperature was set at 70 °C for 2 min, then increased from 70 to 200 °C (3 °C/min) and held at 200 °C for 18 min; the carrier gas was helium (1.0 mL/min). The compounds identification was based on the comparison of their retention indices (RI), determined relatively to the retention times of n-alkanes (C9–C25), with those reported in the literature [43] and those from Wiley 9 (Wiley, New York, NY, USA) and NIST 14 (National Institute of Standards and Technology; Gaithersburg, MD, USA) mass spectral database. The percentage composition of the samples was computed from the GC peak areas using the normalization method (without correction factors). 

#### 4.2.8. Determination of Pesticides and Metals in Thyme Samples

The analysis of pesticides was performed by modified procedures according to national regulations HRN EN ISO 12393-1, 12393-2 and 12393-3: 2013, extraction with petroleum ether/dichloromethane and determination using the GC-ECD Varian CP-3800 instrument (Varian, Inc., Walnut Creek, CA, USA).

Metal trace content was determined according to the HRN EN ISO 14084: 2005 procedure, or by wet sample digestion by HNO_3_ (microwave digestion) with microwave reaction system Multiwave 3000 (Anton Paar GmbH, Graz, Austria). Determination of metals were conducted on the Perkin Elmer AAS Analyst 800 and ICP-MS Perkin Elmer NexION 300X (PerkinElmer, Inc., Waltham, MA, USA), while Hg traces were determined by the Leco AMA254 Hg analyzer (LECO Inc., St. Joseph, MI, USA).

#### 4.2.9. Experimental Design and Statistical Analysis

The experiment was designed in STATGRAPHICS Centurion (StatPoint Technologies, Inc, Warrenton, VA, USA) software and it is presented in Table 9. All experimental results are shown for extracts obtained by HVED and modified conventional extraction (CE) for comparison. Each sample denotes different process parameters, where T stands for thyme, N for nitrogen gas used during HVED treatment and A for argon. Multi-factor categorical design consisted of 12 experimental trials per gas (argon and nitrogen). The three chosen independent variables for HVED assisted extraction were: treatment time (3 and 9 min), voltage applied during HVED (15 kV or 20 kV for argon, and 20 kV or 25 kV for nitrogen) and concentration of ethanol (0%, 25% or 50%). For HVED extraction, numbers 1–12 are the order of conducted treatment, and in CE extraction, 3 and 9 are referred to treatment time while 0, 25, and 50 stands for concentration of an ethanol solvent (%). A total of 30 extracts were prepared in duplicates and all results are given as average ± standard deviation (SD).

Statistical analysis was performed in XLStat (MS Excel 2010). The PCA analysis of theoretical prediction results (using HSPs and COSMO-RS) was performed in XLStat (MS Excel 2010). The PCA was used as a multivariate statistical analysis tool in the processing of the theoretical results to detect qualitative similarities or differences between two different prediction models. Coding of results was performed before modelling with the purpose of uniformed results: 0 (red color in Table 1 and Table 2), 1 (yellow color) and 2 (green color). A PCA model after Varimax rotation was chosen.

ANCOVA was used to analyze the impact of independent variables: treatment time, voltage, ethanol content and treatment type (CE, HVED—nitrogen or HVED—argon) to measured physical and chemical parameters. For UPLC-MS/MS and GC-MS results, all individual (data not shown) and sum of concentrations of all compounds was assessed. The p-values present the statistical significance of each of the factor, and it was significant at *p* ≤ 0.05. 

## 5. Conclusions

In this work the potential of green extraction of BACs from wild thyme by theoretical and experimental approach was demonstrated. The results of experimental study confirmed the potential of HVED for extractions of wild thyme bioactive compounds using water and ethanol. Extraction yield of polyphenols and antioxidants was enhanced with longer treatment time, higher ethanol content and higher voltage, and it was higher for treatment with nitrogen than argon. In average, HVED extraction showed 2.03 higher yield of extraction compared to CE under same extraction conditions (ethanol content and time of extraction), and it was 2.07 times higher for nitrogen and 1.99 times higher for extraction with argon. The main phenolic compound found in wild thyme extracts was rosmarinic acid, while the predominant volatile compound was monoterpenoid phenol carvacrol. Furthermore, experimental results confirmed theoretical results since higher solubility of wild thyme bioactive compounds was found in ethanol than water. However, more accurate results were obtained using COSMO-RS software, compared to HSPs. Wild thyme extracts obtained by HVED are considered as safe, in terms of pesticides and metals levels, and present a high-quality source of valuable BACs for further use in functional food production.

HVED was presented as fast and effective nonthermal technology where thermolabile BACs are being preserved and recovered in high concentrations. Furthermore, HVED has a potential to be scaled-up to industrial level to replace less environmentally acceptable conventional extraction methods. Finally, the results from this study encourage further investigation of HVED as a green extraction method and its comparison with other green extraction methods.

## Figures and Tables

**Table 1:** Relative energy difference (RED) results by Hansen solubility parameters (HSPs) of bioactive compounds from wild thyme for green solvents in comparison with n-hexane.

Solvents	n-Hexane	Ethyl Acetate	Methyl Acetate	Ethyloleate	Ethanol	1-Butanol	Isopropanol	Methanol	Limonene	α-Pinene	Cymene	β-Myrcene	CPME	DMC	MeTHF	Water
**Monoterpenes**																
Carvacrol	2.52	1.21	1.97	1.43	3.43	2.37	2.56	4.58	0.96	1.64	1.29	1.49	1.12	1.76	0.93	9.34
Thymol	2.52	1.21	1.97	1.43	3.43	2.37	2.56	4.58	0.96	1.64	1.29	1.49	1.12	1.76	0.93	9.34
Carene	0.94	1.75	3.05	0.40	4.77	3.64	3.82	5.86	0.72	0.00	0.59	0.32	0.77	2.65	1.18	10.75
α-thujene	1.10	1.70	3.03	0.41	4.73	3.62	3.80	5.82	0.63	0.20	0.40	0.37	0.70	2.56	1.09	10.72
α-terpinene	1.35	1.46	2.66	0.40	4.34	3.21	3.40	5.46	0.30	0.47	0.51	0.38	0.52	2.35	0.87	10.32
δ-terpinene	1.33	1.42	2.63	0.35	4.33	3.20	3.10	5.44	0.34	0.46	0.54	0.34	0.48	2.32	0.84	15.50
**Oxygenated monoterpenes**																
Borneol	2.28	0.91	1.72	1.22	3.33	2.23	2.42	4.46	0.90	1.50	1.30	1.28	0.90	1.63	0.71	9.30
α-terpineol	2.37	0.98	1.68	1.32	3.26	2.15	2.34	4.40	0.97	1.58	1.38	1.37	1.01	1.67	0.82	9.22
α-Cadinol	1.89	1.24	2.35	0.82	3.95	2.85	3.04	5.08	0.32	0.99	0.69	0.86	0.61	2.02	0.67	9.91
Linalool	2.10	1.02	1.86	1.07	3.49	2.37	2.56	4.63	0.73	1.32	1.17	1.11	0.80	1.82	0.72	9.46
Piperitone	1.82	1.18	2.62	0.80	4.22	3.19	3.37	5.28	0.74	1.02	0.63	0.88	0.55	1.86	0.57	10.19
**Sesquiterpenes**																
β-caryophyllene	1.04	1.73	2.97	0.42	4.68	3.54	3.73	5.78	0.61	0.14	0.57	0.33	0.74	2.63	1.15	10.65
Copaene	1.02	1.97	3.29	0.65	4.99	3.86	4.05	4.09	0.83	0.29	0.55	0.59	0.97	2.83	1.36	10.97
α-cubebene	1.17	1.75	2.98	0.50	4.67	3.53	3.72	5.79	0.52	0.25	0.46	0.43	0.77	2.64	1.15	10.64
β-elemene	0.83	1.77	3.12	0.42	5.92	3.73	3.91	4.85	0.87	0.18	0.68	0.35	0.80	2.65	1.21	10.84
β-cadinene	1.10	1.69	2.94	0.40	4.64	3.51	3.69	5.74	0.55	0.18	0.50	0.32	0.70	2.58	1.09	10.62
δ-cadinene	0.93	1.85	3.15	0.50	4.86	3.73	3.91	5.95	0.77	0.10	0.60	0.42	0.86	2.73	1.26	10.84
**Oxygenated Sesquiterpenes**																
Isospathulenol	1.97	1.26	2.25	0.92	3.84	2.73	2.92	4.99	0.39	1.09	0.83	0.95	0.72	2.05	0.76	9.79
**Diterpenes**																
Carnosic acid	2.99	1.69	2.05	1.94	3.29	2.26	2.46	4.48	1.38	2.10	1.72	1.98	1.66	2.09	1.48	9.09
**Triterpenes**																
Rosmarinic acid	4.56	2.95	2.76	3.49	3.11	2.55	2.69	4.20	2.93	3.65	3.18	3.53	3.17	2.85	2.91	8.29
**Flavanons**																
Naringenin	4.86	2.95	2.70	3.76	2.82	2.50	2.60	3.76	3.30	3.98	3.52	3.82	3.39	2.59	3.06	7.92
Eriodictyol	4.53	2.75	2.89	3.44	3.31	2.85	2.98	4.26	2.99	3.64	3.13	3.50	3.08	2.43	2.77	8.55
**Flavonoids**																
Apigenin	4.37	2.78	2.61	3.30	3.07	2.43	2.59	4.19	2.74	3.46	3.01	3.35	2.99	2.74	2.74	8.35
Luteolin	4.06	2.66	2.89	3.01	3.59	2.87	3.04	4.07	2.45	3.13	2.62	3.05	2.72	2.68	2.50	8.98
Thymusin	4.25	2.68	2.57	3.18	3.11	2.44	2.60	4.23	2.63	3.34	2.88	3.23	2.87	2.65	2.62	8.45
Kaempherol-3-glucuronide	5.73	3.62	2.85	4.64	2.17	2.40	2.41	2.85	4.24	4.91	4.53	4.71	4.26	3.10	3.90	6.71
Apigenin 6.8-di-c-glucoside	3.87	2.12	2.42	2.78	3.18	2.52	2.67	4.22	2.35	2.99	2.51	2.85	2.42	1.93	2.11	8.75
**Other Oxygenated**																
Quinic acid	6.23	4.02	2.90	5.20	1.57	2.37	2.27	1.82	4.89	5.51	5.21	5.27	4.82	3.43	4.44	5.70
Caffeic acid	5.94	4.03	3.15	4.87	2.46	2.47	2.59	3.27	4.38	5.09	4.71	4.92	4.53	3.71	4.22	6.66
Protocatechuic acid	5.99	4.01	3.15	4.91	2.41	2.60	2.62	3.16	4.44	5.14	4.75	4.97	4.56	3.62	4.23	6.61
p-coumaric acid	5.25	3.36	2.60	4.18	2.28	2.11	2.18	3.27	3.70	4.40	4.02	4.23	3.84	3.10	3.53	7.14
Vanillin	4.74	2.69	2.47	3.64	2.61	2.34	2.43	3.49	3.25	3.90	3.46	3.71	3.25	2.20	2.89	7.84
Vanillic acid	5.03	3.06	2.42	3.94	2.25	2.04	2.11	3.23	3.48	4.18	3.78	4.00	3.58	2.72	3.25	7.32
**Vitamins (antioxidants)**																
α-tocopherol	1.40	1.53	2.72	0.48	4.39	3.26	3.44	5.51	0.24	0.48	0.44	0.46	0.59	2.40	0.92	10.36
Ascorbic acid	7.84	5.70	4.56	6.79	3.07	3.92	3.83	2.97	6.40	7.07	6.72	6.85	6.41	5.12	6.05	4.79

Relative energy difference (RED): very good solubility 0–1 (green color); medium solubility 1–3 (yellow color); poor solubility > 3 (red color). CPME—cyclopentyl methyl ether, DMC—dimethylcarbonate, MeTHF—2-methyltetrahydrofuran.

**Table 2:** The conductor like screening model for realistic solvents (COSMO-RS) probability of solubility (%) of bioactive compounds from wild thyme for green solvents in comparison with *n*-hexane.

Solvents	n-Hexane	Ethyl Acetate	Methyl Acetate	Ethyloleate	Ethanol	1-Butanol	Isopropanol	Methanol	Limonene	α-Pinene	Cymene	β-Myrcene	CPME	DMC	MeTHF	Water
**Monoterpenes**																
Carvacrol	10.96	100.00	100.00	100.00	100.00	100.00	100.00	100.00	30.90	16.98	34.67	33.11	100.00	100.00	100.00	0.03
Thymol	16.98	100.00	100.00	100.00	100.00	100.00	100.00	70.79	38.90	24.55	45.71	43.65	100.00	100.00	100.00	0.01
Carene	91.54	55.13	37.70	100.00	11.81	23.92	18.75	4.29	99.29	97.71	92.21	93.52	99.45	24.40	95.92	0.00
α-thujene	93.33	51.29	33.88	100.00	10.47	22.39	18.62	3.89	98.40	98.86	89.13	91.20	97.72	21.38	93.33	0.00
α-terpinene	87.10	60.26	41.69	100.00	12.02	23.99	19.05	4.27	100.00	95.50	95.50	97.72	100.00	27.54	100.00	0.00
γ-terpinene	87.10	58.88	41.69	100.00	11.48	22.91	18.20	4.17	99.91	95.50	95.50	97.72	100.00	26.92	98.86	0.00
**Oxygenated monoterpenes**																
Borneol	11.22	100.00	89.13	81.28	85.11	100.00	100.00	41.69	17.78	13.49	16.98	16.60	100.00	38.02	100.00	0.02
α-terpineol	11.22	75.86	57.54	50.12	60.26	87.10	77.62	30.90	20.42	14.45	20.89	20.42	97.72	31.62	100.00	0.02
α-Cadinol	32.35	100.00	81.28	100.00	41.68	70.79	63.09	14.12	42.65	35.48	38.01	37.15	100.00	30.90	100.00	0.00
Linalool	13.18	100.00	93.33	64.57	52.48	70.79	66.07	25.70	25.70	17.38	28.18	26.92	100.00	48.98	100.00	0.01
Piperitone	32.35	100.00	89.12	85.11	91.20	100.00	100.00	51.28	72.44	46.77	87.09	83.17	87.09	70.79	100.00	0.06
**Sesquiterpenes**																
β-caryophyllene	99.95	53.70	33.11	100.00	7.94	18.62	14.12	2.34	100.00	100.00	89.12	89.53	100.00	18.19	100.00	0.00
Copaene	100.00	31.62	17.78	93.32	6.02	15.48	11.22	1.47	93.32	100.00	74.13	75.85	95.49	8.91	89.12	0.00
α-cubebene	100.00	37.15	21.37	97.72	6.30	16.21	11.48	1.62	97.72	100.00	81.28	81.28	100.00	10.96	97.72	0.00
β-elemene	83.17	79.43	54.95	100.00	10.00	19.49	15.48	3.23	100.00	91.20	100.00	99.83	100.00	32.35	100.00	0.00
β-cadinene	100.00	45.70	27.54	100.00	7.07	16.98	12.58	1.86	99.83	100.00	85.11	85.11	100.00	14.45	100.00	0.00
δ-cadinene	100.00	47.86	28.18	100.00	7.76	18.19	13.80	2.18	99.54	100.00	85.11	85.11	100.00	15.13	100.00	0.00
**Oxygenated Sesquiterpenes**																
Isospathulenol	13.48	99.90	69.18	61.65	47.86	75.85	74.13	20.89	21.87	16.21	21.37	20.41	100.00	28.84	100.00	0.00
**Diterpenes**																
Carnosic acid	0.86	100.00	100.00	100.00	100.00	100.00	100.00	100.00	3.37	1.41	3.89	3.49	100.00	100.00	100.00	0.00
**Triterpenes**																
Rosmarinic acid	0.00	100.00	100.00	34.92	100.00	100.00	100.00	100.00	0.07	0.01	0.13	0.11	100.00	100.00	100.00	0.02
**Flavanons**																
Naringenin	0.00	100.00	100.00	76.03	100.00	100.00	100.00	100.00	0.07	0.01	0.12	0.10	100.00	100.00	100.00	0.31
Eriodictyol	0.00	100.00	100.00	100.00	100.00	100.00	100.00	100.00	0.03	0.00	0.05	0.04	100.00	100.00	100.00	0.89
**Flavonoids**																
Apigenin	0.00	100.00	100.00	25.70	100.00	100.00	100.00	100.00	0.02	0.00	0.03	0.03	100.00	100.00	100.00	0.18
Luteolin	0.00	100.00	100.00	54.95	100.00	100.00	100.00	100.00	0.01	0.00	0.02	0.04	100.00	100.00	100.00	0.46
Thymusin	0.02	100.00	100.00	33.11	100.00	100.00	100.00	100.00	0.28	0.06	0.52	0.46	100.00	100.00	100.00	0.00
Kaempherol-3-glucuronide	0.00	100.00	100.00	21.11	100.00	100.00	100.00	100.00	0.00	0.00	0.00	0.00	100.00	100.00	100.00	0.46
Apigenin 6.8-di-c-glucoside	0.00	9.70	17.80	0.00	100.00	100.00	100.00	100.00	0.00	0.00	0.00	0.00	1.63	0.17	100.00	3.04
**Other Oxygenated**																
Quinic acid	0.00	100.00	100.00	0.21	100.00	100.00	100.00	100.00	0.00	0.00	0.00	0.00	80.75	12.09	100.00	66.50
Caffeic acid	0.00	100.00	100.00	0.19	100.00	100.00	100.00	100.00	0.00	0.00	0.00	0.00	100.00	20.89	100.00	85.11
Protocatechuic acid	0.00	100.00	100.00	100.00	100.00	100.00	100.00	100.00	0.03	0.01	0.05	0.04	100.00	100.00	100.00	48.04
p-coumaric acid	0.00	100.00	100.00	13.27	100.00	100.00	100.00	100.00	0.02	0.00	0.03	0.03	100.00	100.00	100.00	2.57
Vanillin	1.07	100.00	100.00	20.89	67.61	50.12	58.88	54.95	4.90	2.09	7.59	7.08	72.44	91.20	100.00	0.17
Vanillic acid	0.01	100.00	100.00	7.94	100.00	100.00	100.00	100.00	0.11	0.03	0.18	0.16	100.00	93.33	100.00	1.29
**Vitamins (antioxidants)**																
Ascorbic acid	0.00	100.00	100.00	0.50	100.00	100.00	100.00	100.00	0.00	0.00	0.00	0.00	100.00	25.91	100.00	100.00
α-tocopherol	66.24	100.00	100.00	100.00	15.19	42.93	33.22	1.86	89.33	67.94	73.87	69.41	100.00	23.24	100.00	0.00

COSMO-RS: Low probability of solubility 0–20% (red color); medium probability of solubility 20–60% (yellow color); high probability of solubility 60–100% (green color). CPME—cyclopentyl methyl ether, DMC—dimethylcarbonate, MeTHF—2-methyltetrahydrofuran.

**Table 3:** Values of pH, conductivity (μS/cm) for CE and HVED treated samples, starting temperature (°C), final temperature (°C), power (kW) and cell disintegration index after HVED treatments.

Sample	pH	Conductivity (μS/cm)	Starting Temperature (°C)	Final Temperature (°C)	Power (kW)	Z_p_	Extraction Type
3 T0	5.91 ± 0.20	621.0 ± 13.2	20.6 ± 0.1	/	/	/	CE
9 T0	5.77 ± 0.17	560.0 ± 22.3	20.6 ± 0.4	/	/	/	
3 T25	6.06 ± 0.29	319.0 ± 12.5	20.9 ± 0.9	/	/	/	
9 T25	6.03 ± 0.23	283.7 ± 2.6	20.9 ± 1.2	/	/	/	
3 T50	6.28 ± 0.34	133.8 ± 12.1	20.2 ± 0.3	/	/	/	
9 T50	6.28 ± 0.18	121.8 ± 9.1	20.4 ± 0.1	/	/	/	
TN1	6.13 ± 0.12	169.0 ± 2.3	23.3 ± 1.3	23.9 ± 1.7	12.0 ± 0.0	0.15	HVED extraction
TN2	5.82 ± 0.71	522.0 ± 12.1	24.7 ± 0.2	27.5 ± 0.5	16.0 ± 1.0	0.28	
TN3	5.82 ± 0.32	566.0 ± 20.4	24.7 ± 0.8	25.3 ± 0.4	14.0 ± 2.0	0.41	
TN4	5.83 ± 0.61	556.0 ± 13.2	25.1 ± 0.7	25.3 ± 1.3	12.0 ± 0.0	0.33	
TN5	6.04 ± 0.19	299.0 ± 12.8	25.0 ± 0.4	25.5 ± 0.5	22.0 ± 1.0	0.15	
TN6	6.02 ± 0.81	256.0 ± 8.1	23.8 ± 0.1	25.1 ± 0.9	12.0 ± 1.0	0.05	
TN7	6.31 ± 0.15	118.0 ± 11.3	22.4 ± 1.1	25.2 ± 1.5	11.0 ± 2.0	0.02	
TN8	6.22 ± 0.33	115.0 ± 12.7	22.8 ± 0.3	24.7 ± 0.6	17.0 ± 0.0	0.01	
TN9	6.08 ± 0.71	263.0 ± 20.1	24.2 ± 0.2	24.5 ± 1.3	17.0 ± 3.0	0.02	
TN10	5.77 ± 0.11	612.0 ± 30.4	24.4 ± 0.6	32.1 ± 2.0	22.0 ± 1.0	0.73	
TN11	6.24 ± 1.02	114.0 ± 12.5	24.6 ± 1.2	25.2 ± 0.6	20.0 ± 1.0	0.06	
TN12	6.15 ± 0.26	169.0 ± 20.1	24.5 ± 2.1	25.1 ± 1.9	12.0 ± 0.0	0.03	
TA1	6.13 ± 0.42	94.7 ± 11.8	25.0 ± 0.8	25.1 ± 2.2	8.0 ± 1.0	0.02	
TA2	5.39 ± 0.38	646.0 ± 30.1	25.6 ± 1.1	37.8 ± 1.3	12.0 ± 2.0	1.00	
TA3	5.40 ± 0.27	590.0 ± 13.7	25.3 ± 0.2	29.0 ± 0.9	9.0 ± 0.0	0.59	
TA4	5.55 ± 0.16	608.0 ± 14.6	25.2 ± 2.0	27.9 ± 1.4	14.0 ± 1.0	0.72	
TA5	5.76 ± 0.21	351.0 ± 13.9	21.3 ± 0.5	31.2 ± 2.3	16.0 ± 2.0	0.28	
TA6	5.50 ± 0.34	280.0 ± 7.1	25.0 ± 0.4	28.5 ± 0.9	10.0 ± 0.0	0.10	
TA7	5.12 ± 0.18	109.6 ± 9.6	23.6 ± 1.7	25.1 ± 1.1	9.0 ± 1.0	0.00	
TA8	6.12 ± 1.12	116.4 ± 11.5	20.4 ± 2.3	27.4 ± 0.8	17.0 ± 2.0	0.06	
TA9	5.88 ± 0.51	246.2 ± 12.4	25.1 ± 0.1	26.1 ± 1.2	14.0 ± 0.0	0.13	
TA10	5.60 ± 0.40	605.0 ± 17.4	23.2 ± 0.4	32.3 ± 0.1	16.0 ± 1.0	0.67	
TA11	6.19 ± 0.18	89.0 ± 6.3	23.6 ± 2.4	24.1 ± 0.2	12.0 ± 1.0	0.01	
TA12	5.91 ± 0.26	222.7 ± 20.3	23.8 ± 1.5	25.2 ± 0.6	9.0 ± 0.0	0.08	

/—not applicable.

**Table 4:** UPLC-MS/MS analysis of extractive compounds from thyme (measurements for CE and HVED treated samples) (ng/mL).

Sample	Concentration (ng/mL)												Extraction Type
	Apigenin	Carnosol	Diosmetin	Hydroxytyrosol	Luteolin	Oleanolic Acid	Quercetin	Rosmarinic Acid	p-Cymene	Thymol	Carvacrol	Camphor	
3 T0	12.114	0.101	31.051	12.543	101.718	4.920	0.220	3611.057	0.147	0.100	0.023	0.497	CE
9 T0	N/D	0.546	N/D	49.436	N/D	58.755	N/D	1.983	1.255	0.023	0.003	1.177	
3 T25	N/D	0.284	3.851	53.034	1.109	N/D	1.073	1050.120	0.018	0.857	0.003	0.145	
9 T25	45.879	0.273	32.355	27.798	226.458	N/D	1.794	6010.666	1.149	0.010	0.098	0.138	
3 T50	66.073	0.354	28.432	14.164	170.078	458.380	1.616	4665.132	0.029	0.041	N/D	10.301	
9 T50	105.846	3.491	54.155	33.044	318.747	810.724	44.725	5906.846	0.926	0.001	0.001	15.601	
TN1	73.998	1.825	66.335	33.949	262.150	345.395	30.299	5156.860	0.122	0.000	0.000	1.178	HVED extraction
TN2	21.900	0.116	35.950	42.139	233.229	7.658	2.328	4291.215	0.038	0.907	0.429	0.134	
TN3	16.618	0.083	35.840	25.589	157.588	3.713	1.456	4393.277	0.001	0.027	0.008	1.860	
TN4	23.733	0.122	39.986	33.498	225.259	4.512	1.580	4586.689	0.037	0.000	0.000	0.017	
TN5	38.606	0.242	32.407	26.316	211.022	3.339	1.884	5047.926	0.007	2.957	0.000	N/D	
TN6	32.662	0.249	29.816	22.327	189.079	N/D	1.088	4722.660	0.002	N/D	0.001	0.056	
TN7	92.601	3.290	66.702	31.581	293.050	559.388	39.952	5294.104	0.048	0.000	0.002	2.243	
TN8	98.111	11.187	63.431	37.787	306.348	1044.092	65.791	5510.994	0.078	0.000	0.000	2.328	
TN9	36.492	0.291	33.546	28.701	207.496	5.163	2.020	5231.490	0.001	0.000	0.000	0.067	
TN10	N/D	0.025	0.088	0.036	0.871	1.106	N/D	42.969	0.028	0.000	0.000	0.628	
TN11	91.870	4.083	64.151	31.035	285.800	561.813	43.709	5319.695	0.000	0.000	0.000	0.045	
TN12	56.268	0.452	39.586	25.776	218.796	19.802	3.778	4843.199	0.009	0.000	0.000	0.016	
TA1	82.733	6.732	59.872	31.115	260.344	869.002	60.758	5367.013	0.054	1.011	2.110	1.519	
TA2	21.497	0.162	128.078	285.617	227.339	5.090	2.631	4342.936	0.057	0.000	0.000	N/D	
TA3	22.760	0.239	39.428	33.397	183.656	5.541	2.097	4649.884	0.009	0.000	0.000	0.010	
TA4	23.128	0.118	44.780	33.985	216.383	12.748	2.016	4701.504	0.001	0.000	0.000	N/D	
TA5	33.864	0.220	30.660	27.678	209.574	3.946	1.579	4711.450	0.006	0.000	0.001	N/D	
TA6	48.515	0.443	43.000	32.625	306.700	3.738	1.772	4890.886	0.131	0.000	0.000	0.025	
TA7	88.655	13.187	55.124	32.586	262.343	699.078	37.623	5314.026	0.102	0.000	0.000	0.041	
TA8	86.062	7.819	58.728	28.403	290.954	755.546	50.080	5199.188	N/D	0.000	0.000	0.023	
TA9	32.634	0.322	26.486	25.103	167.677	4.245	2.197	4882.020	N/D	0.000	0.001	0.218	
TA10	40.040	0.107	74.451	145.722	431.039	10.132	38.575	4767.469	0.099	0.146	0.000	1.458	
TA11	98.464	4.025	52.703	28.518	254.466	818.490	50.960	5097.063	0.060	0.000	0.000	0.038	
TA12	29.757	0.159	27.226	20.202	177.483	3.595	1.150	4825.154	0.045	0.001	0.002	0.356	

N/D—not detected.

**Table 5:** GC-MS analysis of volatile compounds from wild thyme (measurements for CE and HVED treated samples) (%).

Sample	Area (%)				Extraction Type
	1,8-Cineole (RI = 1038)	Linalool (RI = 1103)	Thymol (RI = 1302)	Carvacrol (RI = 1310)	
3 T0	1.40	14.97	9.12	14.78	CE
9 T0	/	0.21	0.48	0.47	
3 T25	/	/	/	/	
9 T25	1.72	15.03	9.43	15.20	
3 T50	/	0.30	0.76	1.21	
9 T50	/	/	/	/	
TN1	/	/	/	/	HVED extraction
TN2	1.41	11.36	9.21	15.30	
TN3	2.30	13.28	10.52	17.52	
TN4	3.46	13.99	10.08	17.70	
TN5	/	/	1.37	2.71	
TN6	/	/	1.14	2.44	
TN7	/	/	/	/	
TN8	/	/	/	/	
TN9	/	/	1.49	2.49	
TN10	0.96	13.00	11.50	21.86	
TN11	/	/	/	/	
TN12	/	/	/	/	
TA1	/	/	/	/	
TA2	1.48	6.01	9.87	17.49	
TA3	1.59	9.90	10.06	17.09	
TA4	1.22	11.44	10.41	17.88	
TA5	/	0.92	2.76	4.33	
TA6	/	0.61	1.86	2.85	
TA7	/	/	/	/	
TA8	/	/	/	/	
TA9	/	/	1.03	1.71	
TA10	1.27	14.42	11.50	20.55	
TA11	/	/	/	/	
TA12	/	/	/	/	

/—not detected, RI—retention index.

**Table 6:** Analysis of covariance (ANCOVA) for pH, conductivity, temperature difference, power, TPC, FRAP, DPPH, yield of extraction and sum of all compounds measured by UPLC-MS/MS and GC-MS methods. The *p*-values present the statistical significance of each of the factors.

	*p*-Value*			
Main Effect	Treatment Time	Voltage	Ethanol Content	Treatment Type
pH	0.709	0.092	<0.0001	0.183
Conductivity	0.447	0.426	<0.0001	0.158
Temperature difference	0.000	0.371	0.017	0.014
Power	0.015	<0.0001	0.290	0.110
Z_p_	0.374	0.619	<0.0001	0.142
TPC	0.125	0.324	0.017	0.735
DPPH	0.023	0.904	0.623	0.463
FRAP	0.358	0.423	0.030	0.531
Yield	0.125	0.324	0.017	0.735
Sum UPLC-MS/MS	0.549	0.977	0.000	0.427
Sum GC-MS	<0.0001	0.006	<0.0001	0.332

* *p* ≤ 0.05 is statistically significant; TPC—total phenolic content, DPPH—2,2-diphenyl-2-picrylhydrazyl free radical assay, FRAP—ferric reducing antioxidant power assay, UPLC-MS/MS—ultra performance liquid chromatography-tandem mass spectrometry characterization, GC-MS—gas chromatography and mass spectrometry analysis.

**Table 7:** Residue levels and maximum residue levels (MRLs) of pesticides (mg/kg) in wild thyme sample.

Pesticides	MRL (mg/kg)	Content (mg/kg)
Alachlor	0.02	<0.005
Aldrin and dieldrin (aldrin and dieldrin combined expressed as dieldrin)	0.01	<0.002
Captan (sum of captan and THPI, expressed as captan)	0.06	<0.020
DDT (sum of p,p′-DDT, o,p′-DDT, p-p′-DDE and p,p′-TDE (DDD) expressed as DDT)	0.05	<0.004
Endosulfan (sum of α- and β-isomers and endosulfan-sulphate expressed as endosulfan)	0.05	<0.002
Endrin	0.01	<0.004
Heptachlor (sum of heptachlor and heptachlor epoxide expressed as heptachlor)	0.01	<0.002
Hexachlorobenzene	0.01	<0.002
Hexachlorocyclohexane, α-isomer	0.01	<0.002
Hexachlorocyclohexane, β-isomer	0.01	<0.002
Iprodione	20.00	<0.010
Lindane (γ-isomer of hexachlorocyclohexane )	0.01	<0.002
Methoxychlor	0.01	<0.010
Tolylfluanid (sum of tolylfluanid and dimethylaminosulfotoluidide expressed as tolylfluanid)	0.05	<0.020
Vinclozolin	0.02	<0.002

MRL—maximum residue level, DDT—dichlorodiphenyltrichloroethane, DDE—dichlorodiphenyldichloroethylene, TDE—tetrachlorodiphenylethane, DDD—dichlorodiphenyldichloroethane.

**Table 8:** Residue levels and maximum residue levels (MRL) of metals (mg/kg) in dried wild thyme and in wild thyme extract.

Metals	MRL (mg/kg)	Dried Wild Thyme (mg/kg)	Extract (mg/kg)
Lead (Pb)	3.000	<0.050	/
Cadmium (Cd)	1.000	0.120	/
Mercury (Hg)	0.100	0.019	/
Chromium (Cr)	/	12.000	56.550
Nickel (Ni)	/	20.060	6.450
Manganese (Mn)	/	115.000	40.750
Iron (Fe)	/	1581.000	25.350
Copper (Cu)	/	8.400	5.700
Zinc (Zn)	/	42.000	13.000

MRL—maximum residue level.

**Table 9:** Denotation of samples, experimental design and process parameters.

Sample	High Voltage Treatment Time (min)	Voltage (kV)	Ethanol Content (%)	Stirring (min)	Extraction Type
3 T0	0	0	0	3	CE
9 T0	0	0	0	9	
3 T25	0	0	25	3	
9 T25	0	0	25	9	
3 T50	0	0	50	3	
9 T50	0	0	50	9	
TN1	3	20	50	0	HVED extraction
TN2	9	20	0	0	
TN3	3	20	0	0	
TN4	3	25	0	0	
TN5	9	25	25	0	
TN6	9	20	25	0	
TN7	9	20	50	0	
TN8	9	25	50	0	
TN9	3	25	25	0	
TN10	9	25	0	0	
TN11	3	25	50	0	
TN12	3	20	25	0	
TA1	3	15	50	0	
TA2	9	15	0	0	
TA3	3	15	0	0	
TA4	3	20	0	0	
TA5	9	20	25	0	
TA6	9	15	25	0	
TA7	9	15	50	0	
TA8	9	20	50	0	
TA9	3	20	25	0	
TA10	9	20	0	0	
TA11	3	20	50	0	
TA12	3	15	25	0	

## Supplementary Materials

The following are available online, Table S1: Correlations between TPC, DPPH, FRAP and sum of all measured compounds by UPLC-MS/MS and GC-MS methods, Figure S1: UPLC-MS/MS chromatograms of main compounds from extract 3 T0: a) apigenin) and b) rosmarinic acid, Figure S2: UPLC-MS/MS chromatograms of main compounds from extract TN8: a) apigenin), b) luteolin, c) rosmarinic acid, and d) oleanolic acid, Figure S3: GC-MS chromatograms of extracts: a) 3 TO, b) TN4, c) TA4 and d) chemical formulas of main compounds found in wild thyme extracts.
