# Supplementary material for: High Voltage Electrical Discharges as an Alternative Extraction Process of Phenolic and Volatile Compounds from Wild Thyme (Thymus serpyllum L.): In Silico and Experimental Approaches for Solubility Assessment
Source: Molecules. 2020 Sep 10;25(18):4131. doi: 10.3390/molecules25184131 (PMC7570489; doi:10.3390/molecules25184131)
Supplement: Supplementary file 1 [file molecules-25-04131-s001.pdf]

Table S1. Correlations between TPC, DPPH, FRAP and sum of all measured compounds by UPLC-MS/MS and GC-MS methods

Correlations	TPC	DPPH	FRAP	SUM UPLC-MS/MS	SUM GC-MS
**TPC**	1				
**DPPH**	0.104	1			
**FRAP**	0.259	0.096	1		
**SUM UPLC-MS/MS**	0.384	-0.048	0.171	1	
**SUM GC-MS**	-0.365	0.098	-0.762	-0.055	1

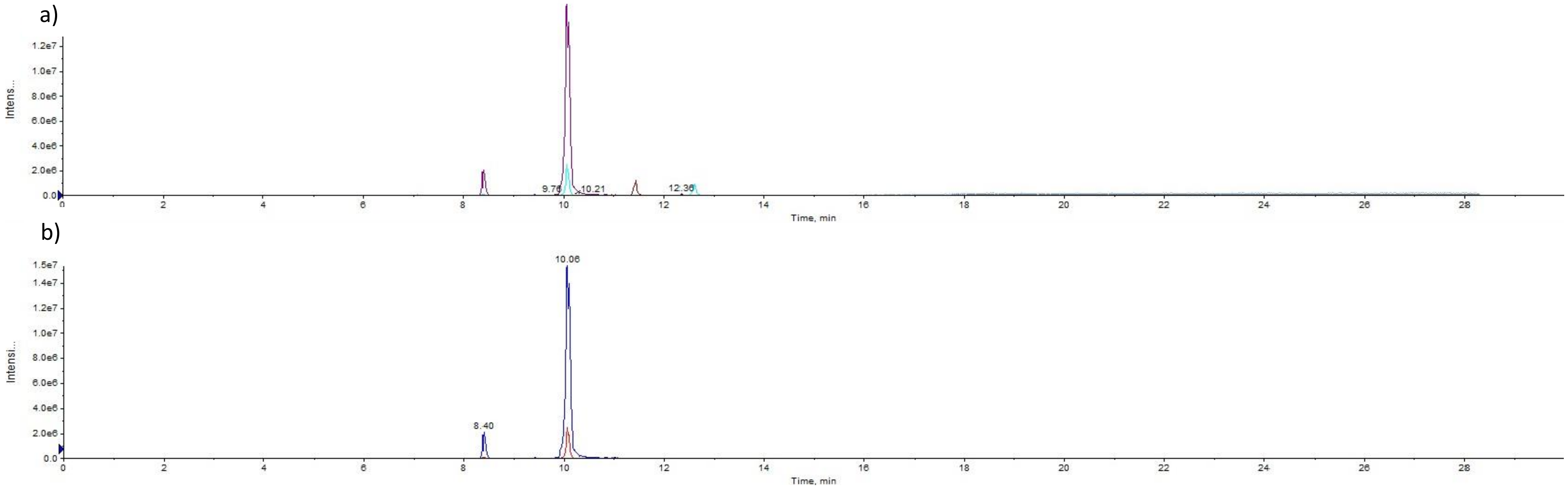

Figure S1. UPLC-MS/MS chromatograms of main compounds from extract 3 T0: a) apigenin) and b) rosmarinic acid

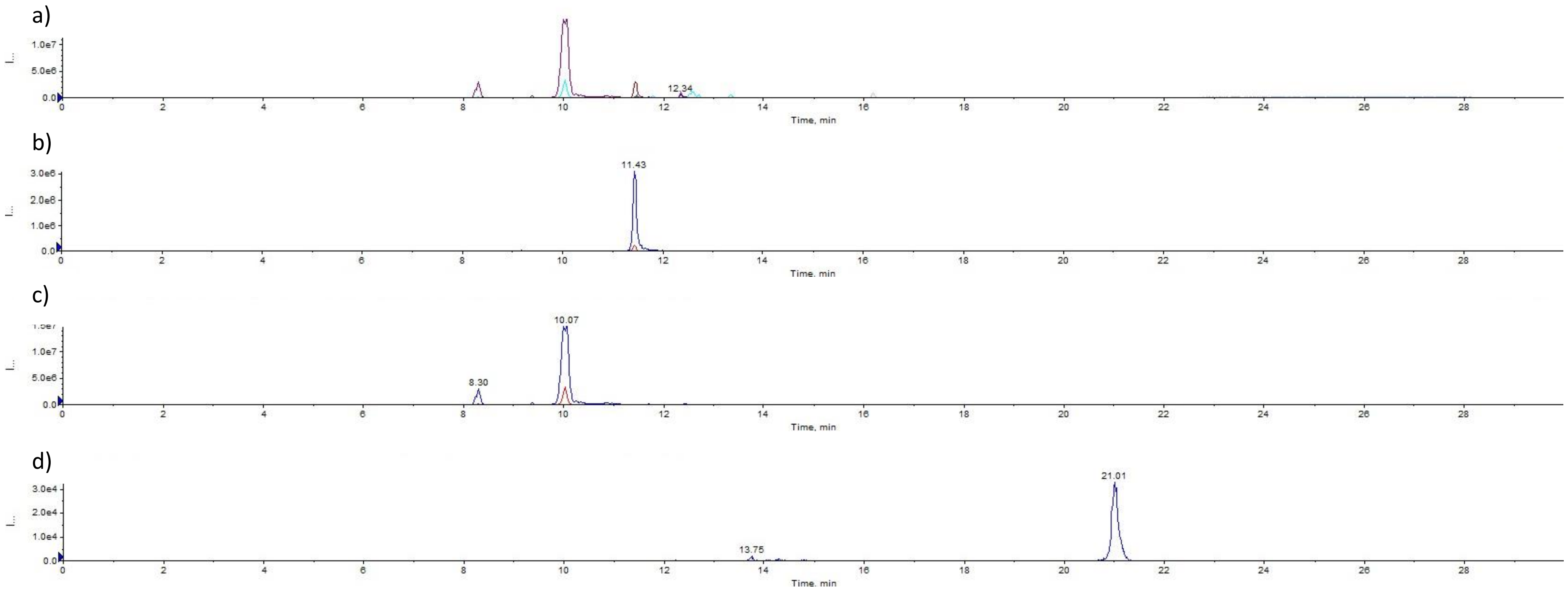

Figure S2. UPLC-MS/MS chromatograms of main compounds from extract TN8: a) apigenin), b) luteolin, c) rosmarinic acid, and d) oleanolic acid

a)

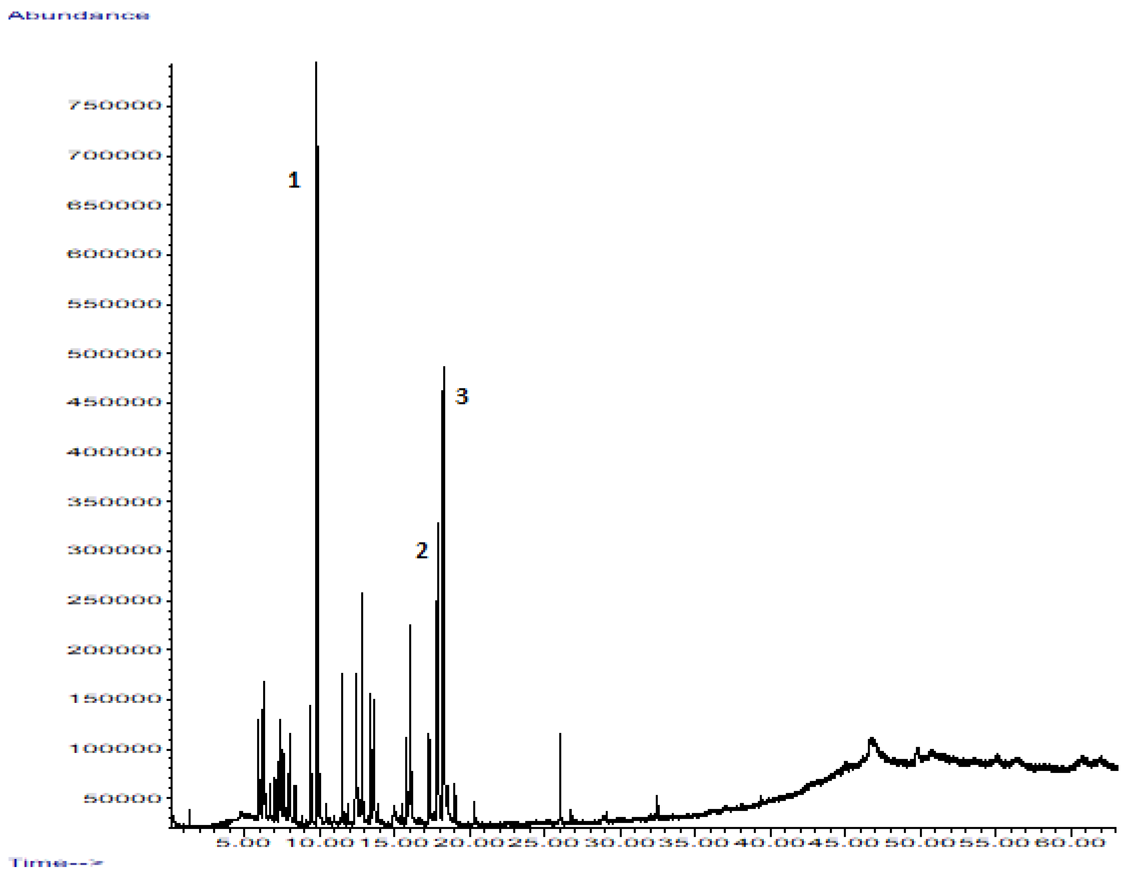
Abundance
750000
700000
650000
600000
550000
500000
450000
400000
350000
300000
250000
200000
150000
100000
50000
1
2
3
5.00
10.00
15.00
20.00
25.00
30.00
35.00
40.00
45.00
50.00
55.00
60.00
Time-->

b)

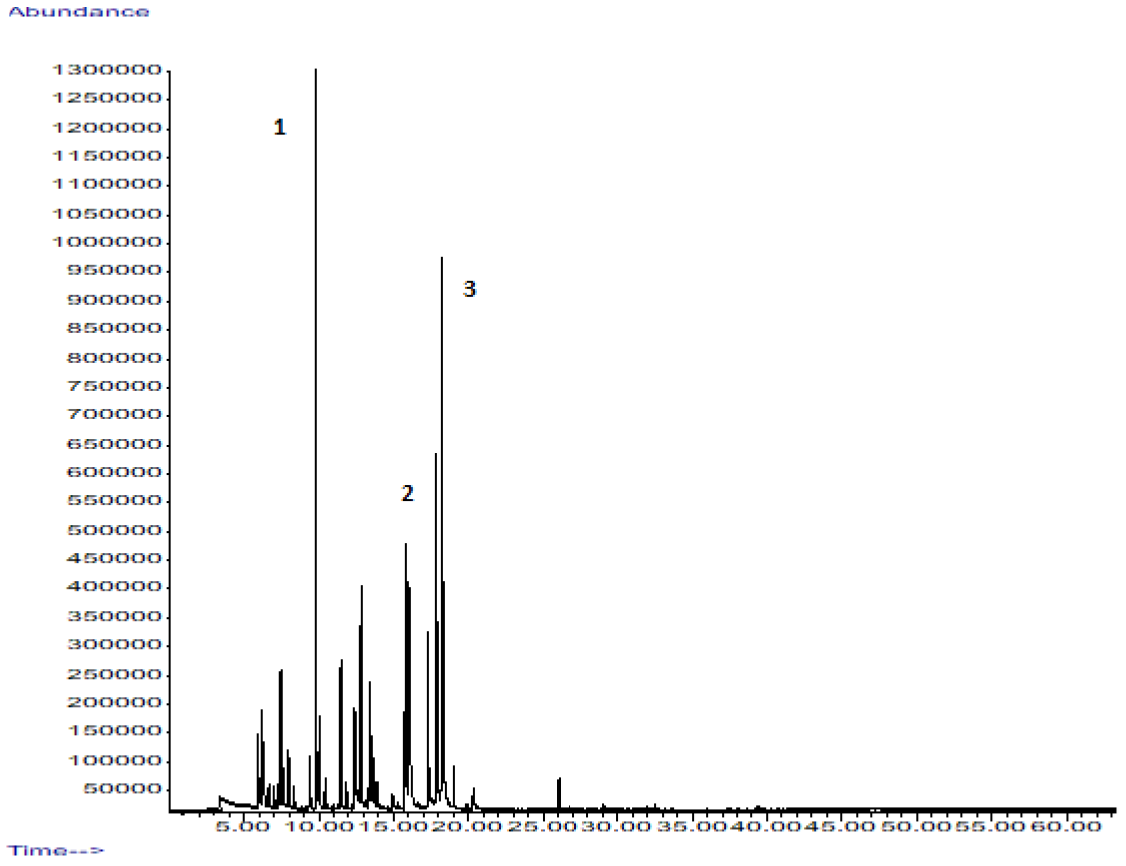
Abundance
1300000
1250000
1200000
1150000
1100000
1050000
1000000
950000
900000
850000
800000
750000
700000
650000
600000
550000
500000
450000
400000
350000
300000
250000
200000
150000
100000
50000
1
2
3
5.00
10.00
15.00
20.00
25.00
30.00
35.00
40.00
45.00
50.00
55.00
60.00
Time-->

c)

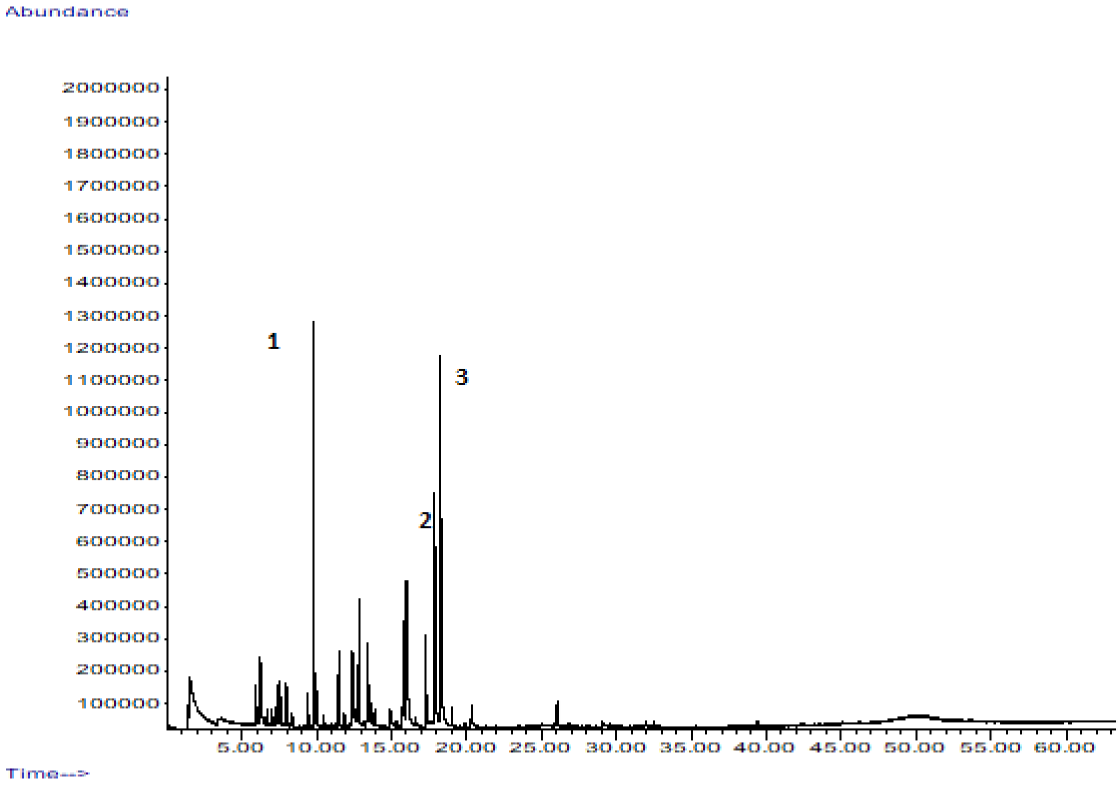

d)

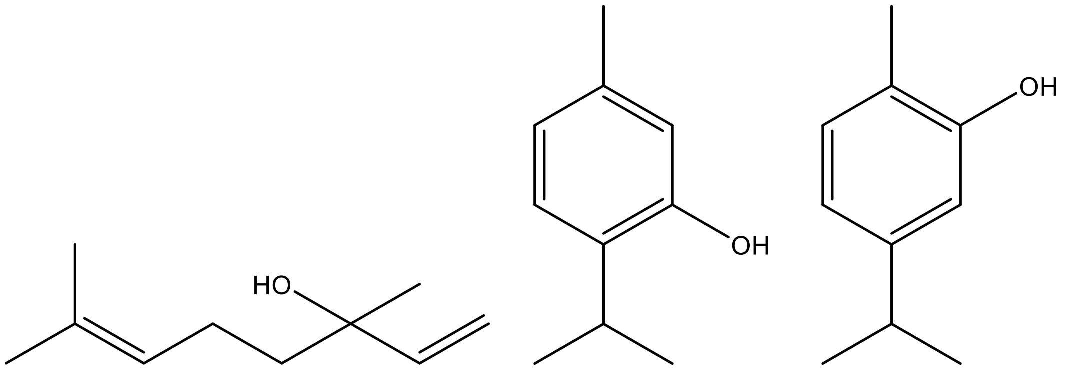

1 – linalool 2 – thymol 3 - carvacrol

Figure S3. GC-MS chromatograms of extracts: a) 3 TO, b) TN4, c) TA4 and d) chemical formulas of main compounds found in wild thyme extracts
